# Integrated CT-derived fractional flow reserve and perivascular fat attenuation index: a multimodal approach to predict in-stent restenosis

**DOI:** 10.3389/fcvm.2025.1703044

**Published:** 2025-12-12

**Authors:** Wentao Zhao, Ling Huang, Mingyuan Zhu, Xingchao Li, Xiaojing Liu, Wanming Zhang, Chunchun Shao, Yixin Li

**Affiliations:** 1 Department of Radiology, The Second Qilu Hospital of Shandong University, Jinan, China; 2 Catheterization Laboratory, The Second Qilu Hospital of Shandong University, Jinan, China; 3 Department of Medical Administration, The Second Qilu Hospital of Shandong University, Jinan, China; 4 Health Management Center, The Second Qilu Hospital of Shandong University, Jinan, China

**Keywords:** computed tomography angiography-derived fractional flow reserve (CT-FFR), pericoronary fat attenuation index (FAI), in-stent restenosis (ISR), multimodal prediction model, predict model

## Abstract

**Background:**

A significant proportion of patients develop in-stent restenosis (ISR) following percutaneous coronary intervention (PCI) with stent implantation, adversely impacting long-term outcomes. Pericoronary fat attenuation index (FAI) and coronary computed tomography angiography-derived fractional flow reserve (CT-FFR) represent emerging non-invasive imaging biomarkers potentially contributing to ISR pathogenesis through inflammatory and hemodynamic mechanisms.

**Objective:**

To construct and evaluate an integrated multimodal predictive model integrating non-invasive computed tomography angiography-derived fractional flow reserve (CT-FFR) and pericoronary fat attenuation index (FAI) for assessing drug-eluting stent (DES)-associated ISR.

**Methods:**

This retrospective cohort study enrolled 144 patients (225 coronary lesions) undergoing coronary CT angiography (CCTA) between 2020 and 2024, followed by PCI within one month and subsequent angiographic follow-up (either invasive coronary angiography or CCTA). Computed tomography angiography-derived fractional flow reserve (CT-FFR) values for target lesions were reconstructed using deep learning algorithms. Pericoronary FAI was quantified at the stented coronary segment. The primary endpoint was angiographically confirmed in-stent restenosis (ISR, defined as luminal stenosis ≥50%) during follow-up. A hybrid-effects logistic regression model generated the integrated CT-FAI-FFR score. Predictive efficacy was evaluated using receiver operating characteristic (ROC) curve analysis and decision curve analysis.

**Results:**

During a median follow-up duration of 25.5 months, ISR occurred in 55 lesions (24.4%). The study cohort was 67.0% male. the multimodal CT-FAI-FFR model achieved an area under the ROC curve (AUC) of 0.793 for predicting ISR. This predictive performance significantly surpassed that of the baseline alone (AUC 0.656, *p* = 0.009).

**Conclusion:**

The CT-FAI-FFR score optimizes ISR risk stratification by integrating complementary information reflecting hemodynamic impairment and local vascular inflammation. This finding suggests a potential pathophysiological interplay between perivascular inflammation and hemodynamic impairment in ISR development.

## 1 Introduction

In the modern era of percutaneous coronary intervention (PCI), in-stent restenosis (ISR) persists as a major clinical challenge, affecting 5%–10% of patients despite the widespread adoption of drug-eluting stents (DES) (1). While anatomical stenosis severity traditionally guides ISR surveillance, an emerging body of evidence underscores the limitations of purely structural assessment. First, approximately 30% of angiographically “mild” lesions (stenoses of 30%–50%) demonstrate hemodynamically significant ischemia (defined by FFR ≤0.80), predisposing them to neointimal hyperplasia (2). Second, pericoronary inflammation—a primary driver of endothelial dysfunction and smooth muscle cell proliferation—remains undetectable through conventional imaging modalities (3, 4). This critical diagnostic gap underscores the compelling need for a pathophysiology-driven, multimodal risk stratification model that synergistically integrates hemodynamic significance and local inflammatory activity to refine ISR prediction.

The Fat Attenuation Index (FAI) represents an emerging biomarker that effectively characterizes both the intensity of vascular inflammation and the progression of atherosclerotic pathology by quantitatively assessing inflammatory activity within pericoronary adipose tissue (PCAT) adjacent to coronary vessels (5, 6). Clinical evidence demonstrates that elevated perilesional FAI values exhibit significant correlation not only with the severity of luminal stenosis, but also display a strong positive association with plaque burden, particularly within non-calcified plaque phenotypes (6, 7). Notably, recent investigations reveal that FAI possesses independent prognostic value for predicting in-stent restenosis (ISR), as confirmed through multivariable regression analyses demonstrating its predictive capacity independent of established clinical risk factors (8). These findings establish FAI as a novel non-invasive imaging biomarker for assessing plaque vulnerability and refining post-procedural risk stratification.

Although the Fat Attenuation Index (FAI) serves as a biomarker reflecting pericoronary inflammation, current research predominantly focuses on its utility in predicting anatomical stenosis following percutaneous coronary intervention (PCI)—a condition primarily driven by inflammation-mediated atherosclerotic plaque progression. Crucially, however, the degree of anatomical stenosis does not equate to hemodynamically significant functional ischemia (9). More importantly, existing in-stent restenosis (ISR) prediction models consistently overlook the pivotal pathophysiological impact of hemodynamic significance, which is closely associated with abnormal wall shear stress. Emerging experimental evidence indicates that LSS triggers inward vessel remodeling through multifaceted mechanisms, including endothelial inflammatory activation, oxidative stress response, extracellular matrix degradation/remodeling, and aberrant smooth muscle cell migration and contraction (10, 11).

Emerging evidence supports the value of combining anatomical and inflammatory biomarkers for ISR risk stratification. For instance, studies by Lin et al. (12) and Dai et al. (13) have demonstrated the prognostic utility of integrating plaque characteristics with pericoronary fat imaging.

Building on this foundation, herein, we propose a novel multimodal ISR prediction model integrating noninvasive CT-derived fractional flow reserve (CT-FFR) with the Fat Attenuation Index (FAI). Our central hypothesis posits that lesion-specific flow impairment quantified by CT-FFR and peri-adventitial inflammatory activity reflected by FAI exhibit interactive effects. Consequently, the multimodal CT-FAI-FFR Score—synthesizing hemodynamic and inflammatory pathway data—was evaluated for significantly enhanced predictive performance for in-stent restenosis (ISR) compared to single-parameter models.

## 2 Methods

### 2.1 Study design and patient selection

This single-center retrospective cohort study, approved by the Institutional Ethics Committee with waived written informed consent, consecutively enrolled patients meeting specific criteria between May 2020 and August 2024. To mitigate the potential confounding effects of medication on the Fat Attenuation Index (FAI), we excluded patients receiving high-dose statin therapy (14) (atorvastatin ≥40 mg/day or equivalent) or systemic anti-inflammatory therapy during the interval between their coronary computed tomography angiography (CCTA) and percutaneous coronary intervention (PCI). All PCI procedures utilized second-generation drug-eluting stents.

**Figure 1:**
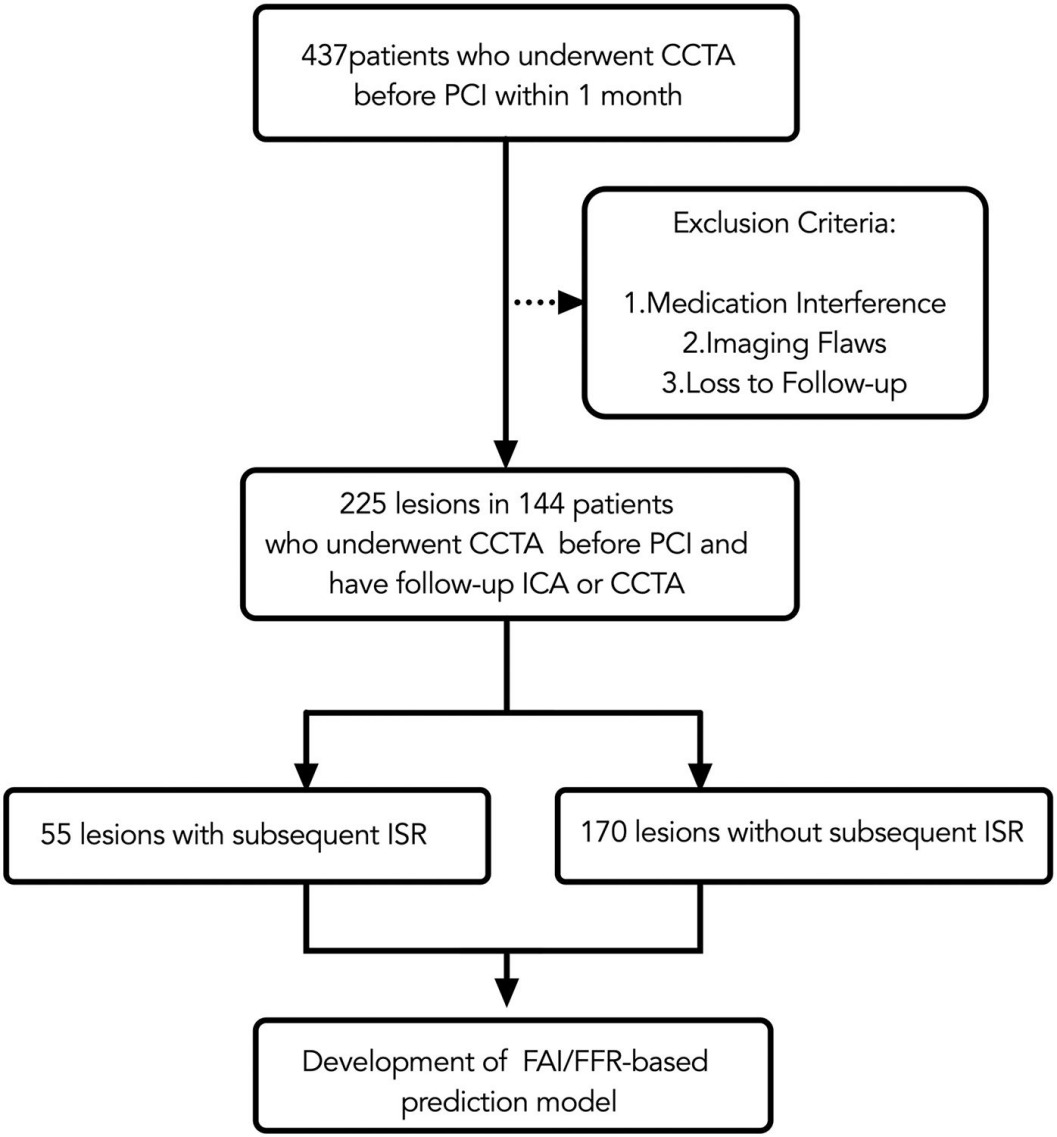
Flow chart. CCTA, coronary CT angiography; PCI, percutaneous coronary intervention; ICA, invasive coronary angiography; ISR, in-stent restenosis; FAI, fat attenuation index.

Final inclusion required patients to fulfill two specific conditions: having a CCTA performed no more than 30 days prior to PCI, with images meeting the quality requirements for both CT-FFR and FAI analysis; and completing follow-up with either invasive coronary angiography (ICA) or diagnostic CCTA for in-stent restenosis (ISR) assessment within 6 to 24 months post-PCI. Exclusion criteria were applied as follows (as summarized in Figure 1): 98 patients (22.4%) for medication interference (high-dose statins or anti-inflammatory therapy), 63 patients (14.4%) for poor image quality [e.g., uncorrectable motion artifacts, severe calcification (Agatston score ≥400) (15), or stent diameters below the reliable assessment threshold of current CCTA guidelines], and 58 patients (13.3%) were lost to follow-up because a lack of standardized imaging or missing clinical endpoint data.

Follow-up imaging with either ICA or CCTA was scheduled for all patients within a pre-specified window of 6 to 24 months post-PCI as part of a standardized surveillance protocol, irrespective of clinical symptoms. For ISR-positive lesions, follow-up was by ICA in 87.3% (48/55) of cases and by CCTA with subsequent ICA confirmation in 12.7% (7/55). For ISR-negative lesions, follow-up was by ICA in 77.6% (132/170) and by CCTA in 22.4% (38/170).

### 2.2 Definition of endpoint

Multiple studies have validated the accuracy of CCTA in diagnosing coronary ISR (16, 17). The primary endpoint is the incidence of ISR, defined as a diameter stenosis of ≥50% detected during follow-up ICA or CCTA (18). For patients who underwent multiple follow-up ICA or CCTA examinations, the latest angiographic findings were used to determine ISR. Follow-up ICA results were retrospectively interpreted by a cardiac interventional specialist (Wanming Zhang, 15 years'experience of PCI interpretation), while follow-up CCTA scans were reviewed retrospectively by a radiologist (Yixin Li, 19 years' experience of CCTA interpretation). Neither the cardiologist nor the radiologist was aware of the clinical information or outcomes related to FAI.

### 2.3 CT-FFR and pericoronary FAI measurement

All coronary computed tomography angiography (CCTA) examinations were performed in accordance with the guidelines established by the Society of Cardiovascular Computed Tomography (SCCT) (19). Following anonymization, image datasets were uniformly imported into the Shukun Coronary Analysis System (v3.0; Shukun Technology Co., Ltd., Beijing; https://www.shukun.net), also commercially referred to as the “coronary doc” or “Careverse FFR” platform, for blinded analyses. This fully automated, deep learning-based system has been clinically validated in large-scale multicenter trials, demonstrating strong agreement with invasive FFR (iFFR) (20) and a significant reduction in unnecessary invasive coronary angiography procedures (21).

Two experienced cardiovascular imaging physicians (H. Zhong and G. Pang, with 23 and 16 years of CCTA interpretation experience, respectively) performed all measurements independently. Both readers were fully blinded to clinical outcomes, patient identities, and each other's measurements. Cases were randomly assigned to the readers using a computer-generated sequence to avoid systematic bias.

When initial independent measurements for CT-FFR or FAI differed beyond pre-defined thresholds (CT-FFR difference >0.05 or FAI difference >5 HU), the readers conducted a structured consensus process: they jointly re-examined the images to identify anatomical landmarks, used manual correction features in the Shukun software to adjust luminal contours if needed, and discussed until a consensus value was reached, which was then used for final analysis.

For subsequent percutaneous coronary intervention (PCI) target lesions, CCTA images were matched with invasive coronary angiography (ICA) datasets by matching anatomical landmarks within the relevant coronary segments. Peri-coronary fat attenuation index (FAI) measurement values were calibrated within the range of −190 to −30 HU, which was validated for clinical use through dedicated studies on the Shukun platform.

**Figure 2:**
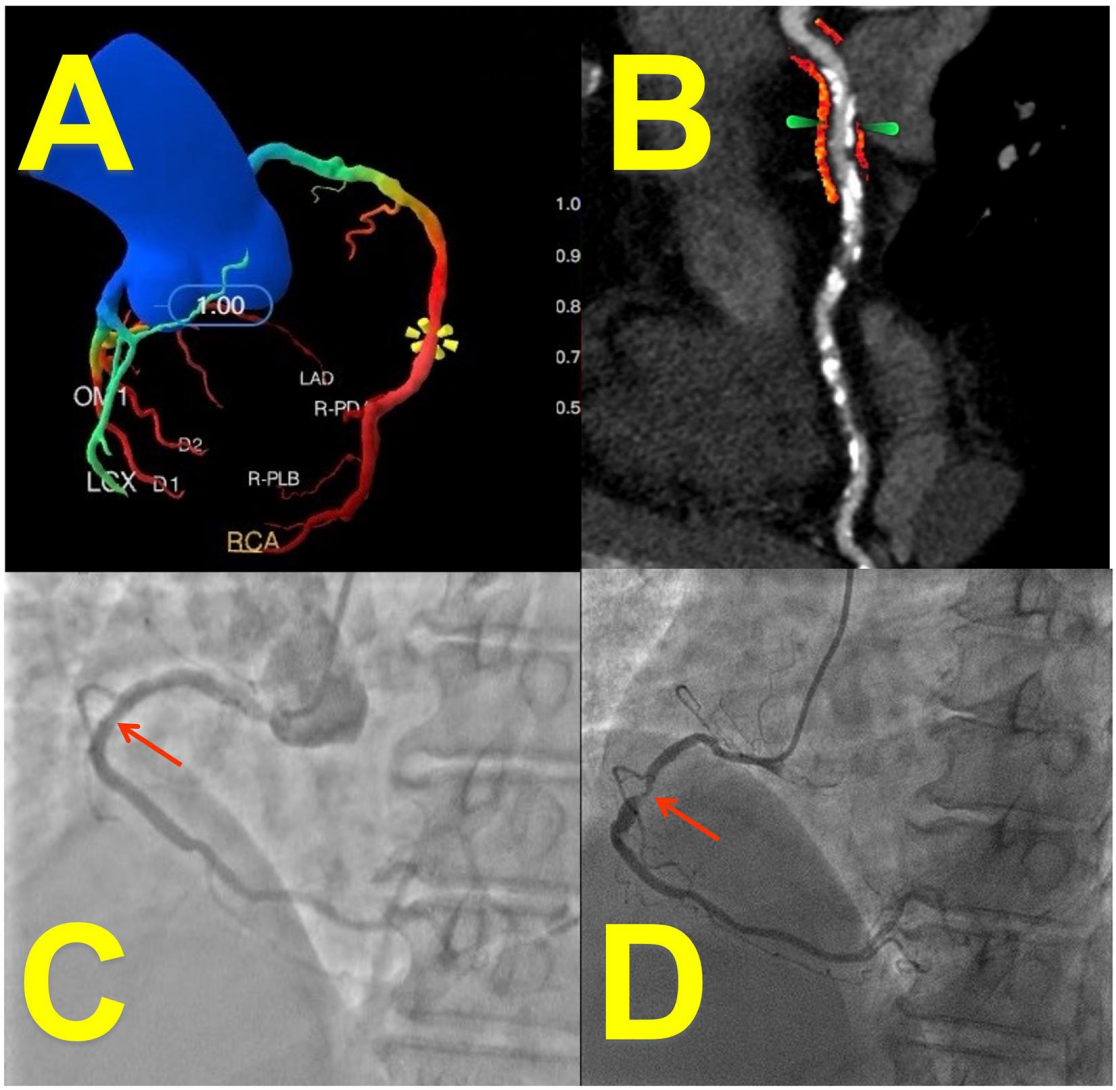
Case presentation: imaging of a 65-year-Old female with coronary artery disease. **(A)** CT-derived FFR (CT-FFR) measured at stenosis of right coronary artery (RCA): 0.58 (deep-learning reconstruction). **(B)** Peri-coronary fat attenuation index (FAI, arrow; red area: fat within −190 to −30 HU range) at stent site: −67 HU (curved-planar CCTA reconstruction). **(C)** Patent stent lumen confirmed on angiography 1 month post-PCI. **(D)** Invasive coronary angiography (ICA) at 16-month follow-up revealed significant in-stent restenosis (ISR, arrow) corresponding to the patient's recurrent exertional chest tightness (relieved within 5 min of rest).

For the analysis of stented vessel segments, the perivascular fat attenuation index (FAI) was quantified on pre-percutaneous coronary intervention (PCI) coronary computed tomography angiography (CCTA) datasets. The stent location on CCTA was identified by matching anatomical landmarks (e.g., side branches, calcifications) between the CCTA and invasive coronary angiography (ICA) to ensure precise segment alignment. During analysis, fiducial markers were placed 5 mm proximal and distal to the intended stent location. The system automatically delineated luminal contours, allowing manual correction when significant calcification impaired image quality. FAI quantification was performed within a region of interest (ROI) defined as a cylindrical volume extending radially outward from the outer vessel wall to a distance equivalent to 1.2 times the mean vessel diameter. This process generated color-coded fat attenuation maps (e.g., Figure 2B), enabling visual verification of measurement accuracy by the interpreters.

Computation of CT-derived fractional flow reserve (CT-FFR) was executed using the Shukun CT-FFR system. The underlying artificial intelligence algorithm is trained on computational fluid dynamics (CFD) models, ensuring its reliability as a non-invasive surrogate for iFFR (22). This involved fully automated extraction of a complete coronary artery model, spanning from the aortic root to distal branches exceeding 1.5 mm in diameter. In regions severely affected by calcification (15) (Agatston score >400) or substantial motion artifacts (e.g., mid right coronary artery), luminal contours underwent manual adjustment by two dedicated cardiovascular imaging physicians (each with >5 years of experience). Hemodynamically significant ischemia was defined by a CT-FFR value ≤ 0.80, with the system automatically highlighting these low-flow territories in red (e.g., Figure 2A). For CT-FFR values falling within the borderline range (0.75–0.85), measurements were repeated three times with the median value used for analysis.

To assess intra- and inter-observer variability, a randomly selected subset of 50 lesions underwent repeat measurements. Intra-observer variability was assessed by having the primary reader (Yixin Li) re-analyze the same cases in a blinded fashion four weeks later. Inter-observer variability was evaluated by having both readers (H. Zhong and G. Pang) independently analyze the same subset of lesions. The intraclass correlation coefficient (ICC) was calculated for both measures, demonstrating excellent agreement (ICC >0.88 for all parameters).

### 2.4 Statistical analysis

All statistical analyses were performed using RStudio (RRID:SCR_000432). Missing data were addressed through multiple imputation using chained equations with 5 iterations (23), creating 10 imputed datasets. Continuous variables were handled using predictive mean matching while categorical variables used logistic regression imputation. Patient-level random effects were incorporated via mixed-effects models to account for correlated lesions within patients. Model adjustments incorporated established ISR risk factors and potential FAI confounders, including:

① Demographic variables (age, gender);
② Cardiovascular risk factors (hypertension, diabetes, smoking, alcohol);
③ Procedural characteristics (minimum stent diameter, stent length);
④ Biomarkers (BNP, Troponin, hsCRP, Glucose, Triglycerides);
⑤ CT-derived indices [FAI, FFR, modified Duke CAD index (24)].

The modified Duke CAD index act as a comprehensive marker of overall plaque burden and anatomical complexity.

To evaluate predictive performance while accounting for intra-patient lesion clustering, we implemented a stratified group repeated cross-validation framework, 50 iterations of random stratification were performed to ensure robustness.

Three sequential logistic regression models were developed:

- Model 1 (Clinical): Age, Gender, Hypertension, Diabetes, BMI, Smoking
- Model 2 (Model 1 + FAI): Integrated pericoronary fat attenuation index
- Model 3 (Model 2 + FFR): Added fractional flow reserve

Mixed-effects modeling with random patient-level intercepts accounted for lesion clustering. Continuous variables were standardized (z-score) to improve convergence.

Discriminatory performance was assessed using receiver operating characteristic (ROC) curves and area under the curve (AUC) comparisons via DeLong's test (25). Calibration curves with grouping by quantiles evaluated prediction accuracy across risk strata (26).

## 3 Results

### 3.1 Baseline characteristics

This study initially included 437 patients who underwent coronary computed tomography angiography (CCTA) within one month prior to percutaneous coronary intervention (PCI). After applying stringent exclusion criteria (medication interference, image quality deficiencies, loss to follow-up), the final analytical cohort comprised 225 coronary lesions from 144 patients. All lesions underwent pre-PCI CCTA, with post-procedural follow-up conducted via invasive coronary angiography (ICA) or repeat CCTA. All PCI procedures utilized second-generation drug-eluting stents with a median diameter of 3.0 mm (IQR: 2.75–3.5 mm) and a median length of 28 mm (IQR: 22–35 mm), confirming that the analysis focused on stents within the validated size range for CCTA assessment. The flow chart is presented in Figure 1.

The study cohort comprised 225 coronary lesions, contributed by 144 patients who underwent coronary computed tomography angiography (CCTA) and were included for model development. The baseline demographic, clinical, cardiovascular risk factors, and CCTA-related characteristics are summarized in Table 1.

**Table 1:** Clinical characteristics and CCTA variables.

Clinical characteristics and CCTA variables	Analyzed lesions (*n* = 225)
Age	61.2 ± 9.7
BMI	26.7 ± 4.1
hsCRP	1.70 (1.00–3.80)
Men	150/225 (67.0%)
Risk faotors	
Hypertension	153/225 (68.9%)
Diabetes	87/225 (39.2%)
Smoking	84/225 (37.8%)
Alcohol	71/225 (32.0%)
Tube voltage	
100 kv	21/225 (9.4%)
120 kv	200/225 (89.7%)
140 kv	2/225 (0.9%)
Equipment	
GE DISCOVERY CT750	22/225 (9.9%)
GE Quark CT	13/225 (5.8%)
GE REVOLUTION	188/225 (84.3%)
Modified duke CAD index	
1	45/225 (28.1%)
2	41/225 (25.6%)
3	41/225 (25.6%)
4	27/225 (16.9%)
5	4/225 (2.5%)
6	2/225 (1.2%)

Values are mean ± SD, *n* (%), or median (IQR).

BMI, body mass index; CAD, coronary artery disease; CCTA, coronary CT angiography; hsCRP, high-sensitivity C-reactive protein.

The study population had a mean age of 61.2 ± 9.7 years and a mean body mass index (BMI) of 26.7 ± 4.1 kg/m^2^, placing the cohort overall in the overweight category. The median high-sensitivity C-reactive protein (hsCRP) level was 1.70 mg/L (interquartile range: 1.00–3.80 mg/L), indicating a degree of systemic inflammation. The majority of participants were male (67.0%, 150/225).

Regarding cardiovascular risk factor burden, hypertension was the most prevalent condition, affecting 68.9% (153/225) of the study population. Diabetes mellitus was also highly prevalent, present in 39.2% (87/225) of patients. Current smokers and alcohol consumers constituted 37.8% (84/225) and 32.0% (71/225) of the cohort, respectively. Most CCTA examinations were performed at a tube voltage of 120 kV (89.7%, 200/225) and conducted primarily on a GE REVOLUTION scanner (84.3%, 188/225). Notably, the modified Duke coronary artery disease (CAD) index exhibited a broad distribution (Class 1: 28.1%; Class 2: 25.6%; Class 3: 25.6%), indicating high lesion complexity.

### 3.2 Incidence and follow-up duration

55 lesions (24.4%) demonstrated in-stent restenosis (ISR). The average time between the initial PCI procedure and follow-up imaging (ICA or CCTA) was 25.5 months (±14.2) for all patients. At the lesion level, follow-up timing was similar between groups: the ISR-positive group was reassessed after 27.3 months (±12.2) and the ISR-negative group after 25.3 months (±11.7), with no statistically significant difference (*p* = 0.587).

The indication for follow-up imaging was protocol-driven rather than symptom-dependent. Among the 144 patients, 68 (47.2%) reported symptoms at follow-up, including chest pain (CCS class I–II, *n* = 52) and dyspnea (*n* = 16). There was no significant difference in the prevalence of symptoms between the ISR and non-ISR groups (*p* = 0.21), supporting the use of a uniform follow-up interval.

### 3.3 Multivariable predictors of in-stent restenosis

**Figure 3:**
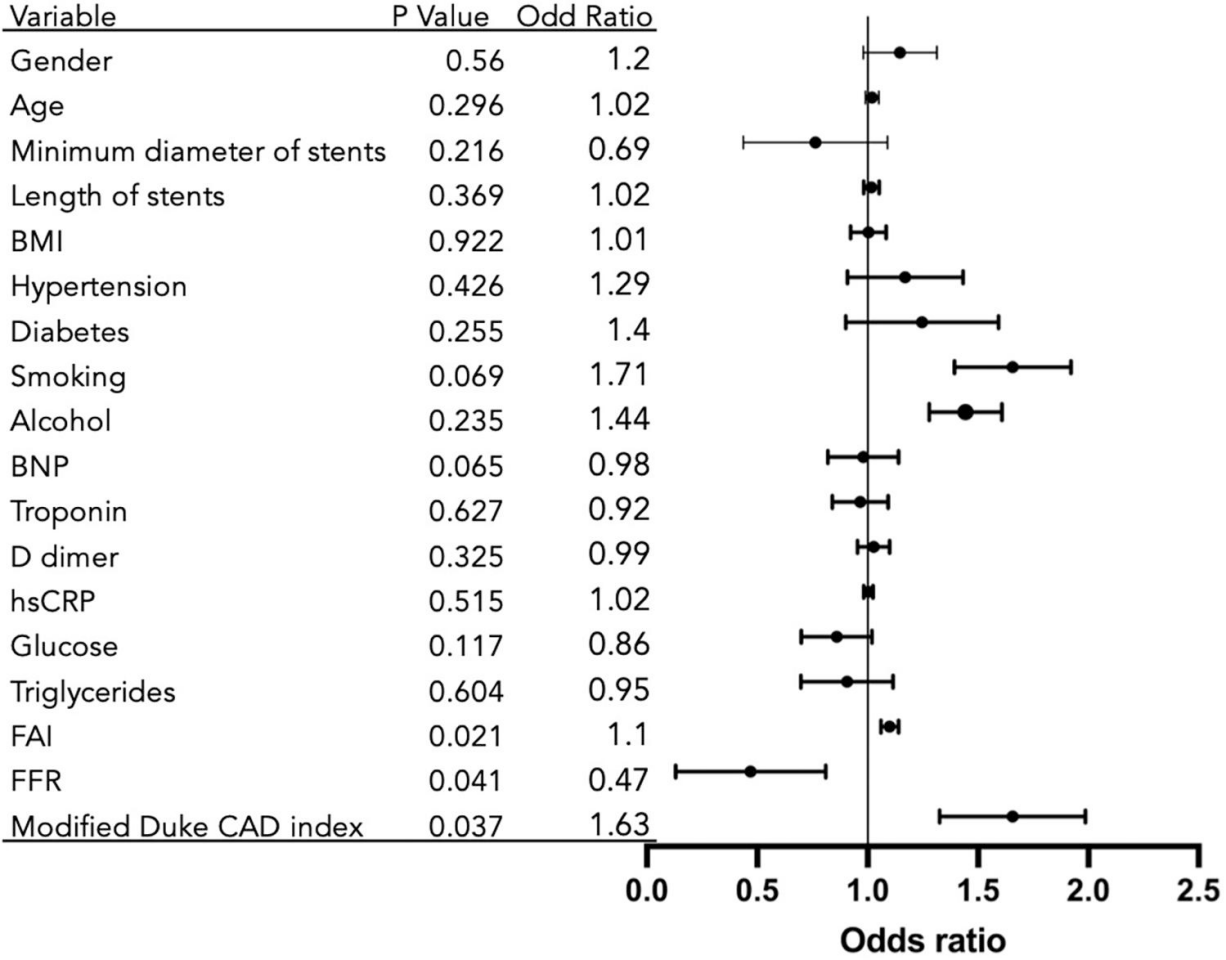
Predictors of in-stent restenosis from multivariable analysis. Variables showing directional trends: FFR: Associated with risk reduction (OR = 0.47; *p* = 0.041); Modified Duke CAD Index: Associated with Increased risk (OR = 1.63; *p* = 0.037); FAI: Suggestive of risk elevation (OR = 1.1; *p* = 0.021); Reference: OR = 1 (no effect).

Multivariable mixed-effects logistic regression analysis, incorporating clinical baseline characteristics, perivascular fat attenuation index (FAI), and fractional flow reserve (FFR), identified significant predictors associated with the risk of in-stent restenosis (ISR), as visualized in Figure 3.

The fat attenuation index (FAI) showed a positive trend with ISR risk (*p* = 0.021). Each 1-unit increase in FAI was associated with a 10% elevated risk of ISR [Odds Ratio [OR] = 1.10; 95% Confidence Interval [CI], 1.06–1.14]. The modified Duke Coronary Artery Disease (CAD) index also suggested a positive association with ISR risk (*p* = 0.037), with each 1-grade increase in the index corresponding to a 63% higher risk of ISR (OR = 1.63; 95% CI, 1.34–2.01).

FFR was indicative of an inverse relationship with ISR risk (*p* = 0.041). A 1-unit decrease in FFR was associated with an odds ratio of 0.47 (95% CI, 0.13–0.81), indicating substantially and significantly increased odds of ISR with worsening hemodynamic impairment. Current smoking status showed a clinically relevant positive association with ISR risk that approached but did not reach statistical significance (OR = 1.71; *p* = 0.069). A case of low FFR combined with elevated FAI value and follow-up found in-stent restenosis is shown in Figure 2.

Several variables showed directional trends that did not achieve statistical significance, including minimum stent diameter (OR = 0.69; *p* = 0.216) and B-type natriuretic peptide (BNP) (OR = 0.98; *p* = 0.065). Multiple other clinical, biochemical, and stent parameters—including gender, age, stent length, BMI, hypertension, diabetes, alcohol consumption, troponin, D-dimer, high-sensitivity C-reactive protein (hsCRP), glucose, and triglycerides—demonstrated no statistically significant associations with ISR risk in this adjusted model (all *p* > 0.05).

### 3.4 Model performance: ROC analysis

Based on clinical and imaging data from the 225 lesions, we developed 3 ISR predictive models. ROC curve analysis demonstrated that the base model (containing clinical variables only) had moderate discriminative ability (AUC = 0.656). Incorporating FAI significantly enhanced model performance (AUC = 0.740; ΔAUC =  + 0.084), achieving good diagnostic accuracy. The combined model integrating FFR (Clinical + FAI + FFR) demonstrated the optimal performance (AUC = 0.793). This finding was corroborated by internal validation, where the mean AUC from 50 iterations of cross-validation was 0.781 (95% CI: 0.752–0.810), indicating stable model performance.

**Figure 4:**
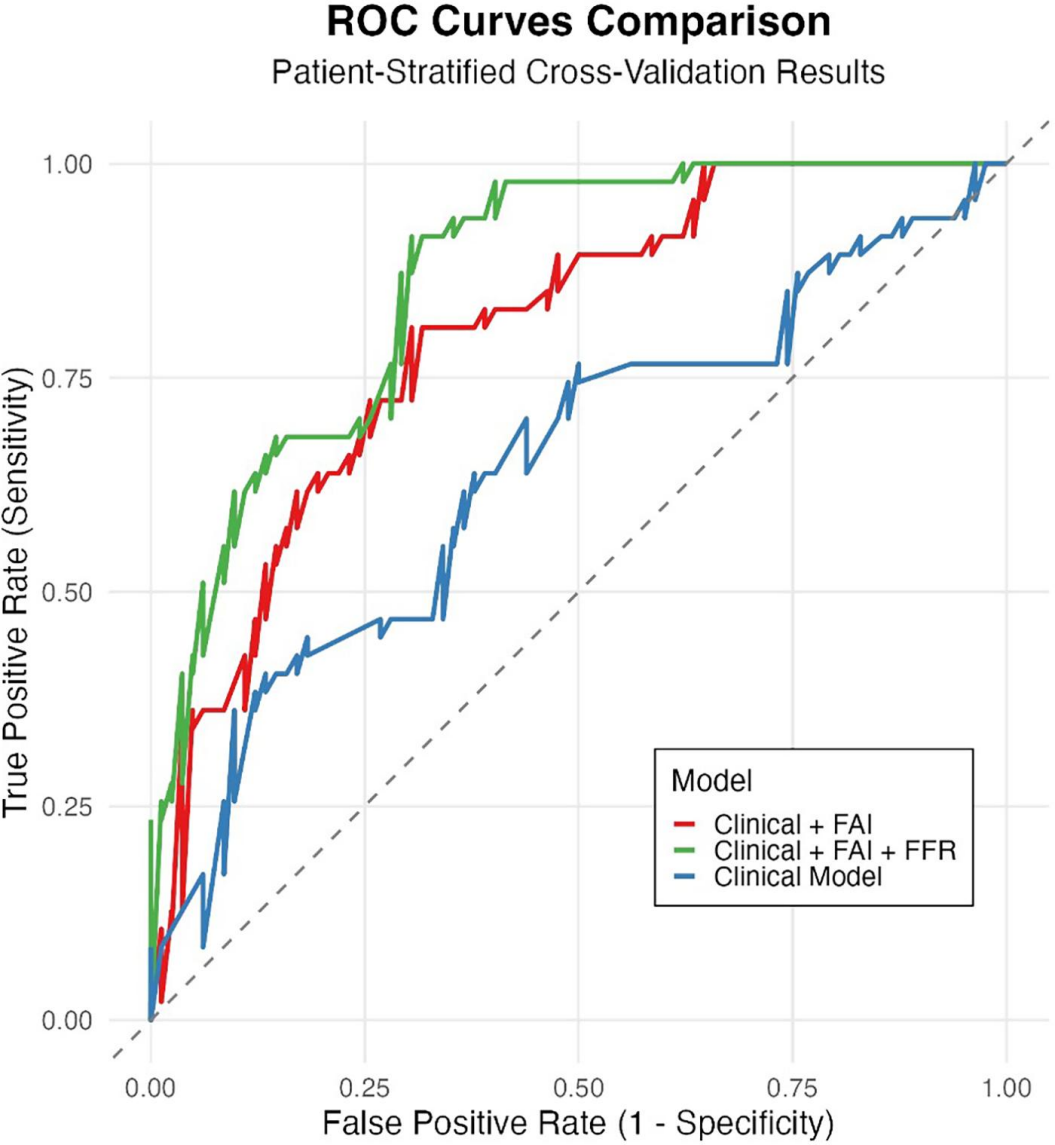
Incremental value of FAI and FFR in predicting ISR risk. Receiver Operating Characteristic (ROC) curves comparing the discrimination performance of three nested models evaluated through patient-stratified cross-validation: Base model (clinical variables only, AUC = 0.656, blue), Base model + Fat Attenuation Index (FAI) (AUC = 0.740, orange), and Base model + FAI + Fractional Flow Reserve (FFR) (AUC = 0.793, green). The diagonal dashed line represents random guessing (AUC = 0.5). Adding FAI significantly improved performance over the Base model, and further adding FFR yielded the highest AUC, demonstrating their additive predictive value.

Visual inspection of ROC curves (Figure 4) confirmed these patterns: the Clinical + FAI + FFR model (green curve) achieved optimal positioning near the top-left corner, followed sequentially by Clinical + FAI (red) and Base (blue) models. Progressive upward-leftward curve shifts with each predictor addition visually validated the stepwise enhancement in risk stratification.

### 3.5 Decision curve analysis

**Figure 5:**
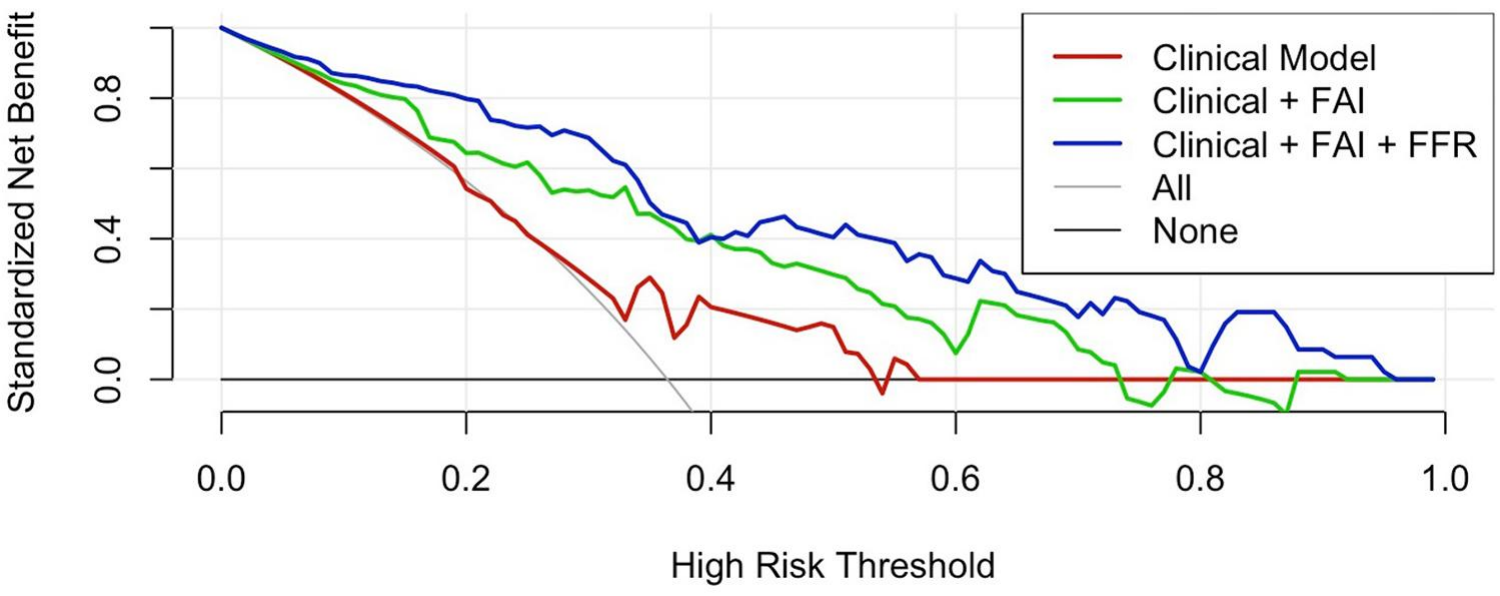
Decision curve analysis. The integrated model demonstrates superior net benefit (0.05–0.78) at clinically relevant thresholds (0.15–0.70), outperforming both comparator models and reference strategies (“ treat-all” and “ treat-none”). Between 0.25–0.65 thresholds, the integrated model exceeded the “treat-all” strategy by 0.12–0.32 points. FAI, Fat attenuation index; FFR, Fractional flow reserve.

Decision curve analysis (Figure 5) revealed significant differences in clinical utility among predictive models across high-risk probability thresholds (0–1.0). The integrated Clinical + FAI + FFR model consistently demonstrated superior standardized net benefit (0.05–0.78) within the clinically relevant threshold range of 0.15–0.70. This comprehensive approach outperformed both the clinical-only model (peak net benefit: 0.34) and the Clinical + FAI hybrid model (peak net benefit: 0.58), with particularly pronounced advantages between thresholds of 0.25–0.65 where it exceeded the net benefit of the “treat-all” strategy by 0.12–0.32 points. At commonly employed decision thresholds (e.g., 0.35), net benefit values progressively improved with each added parameter category: 0.62 (clinical), 0.71 (Clinical + FAI), and 0.78 (Clinical + FAI + FFR). Critically, the composite model maintained clinical superiority over both “treat-all” and “treat-none” approaches beyond the 0.20 threshold.

### 3.6 Calibration performance of predictive models

**Figure 6:**
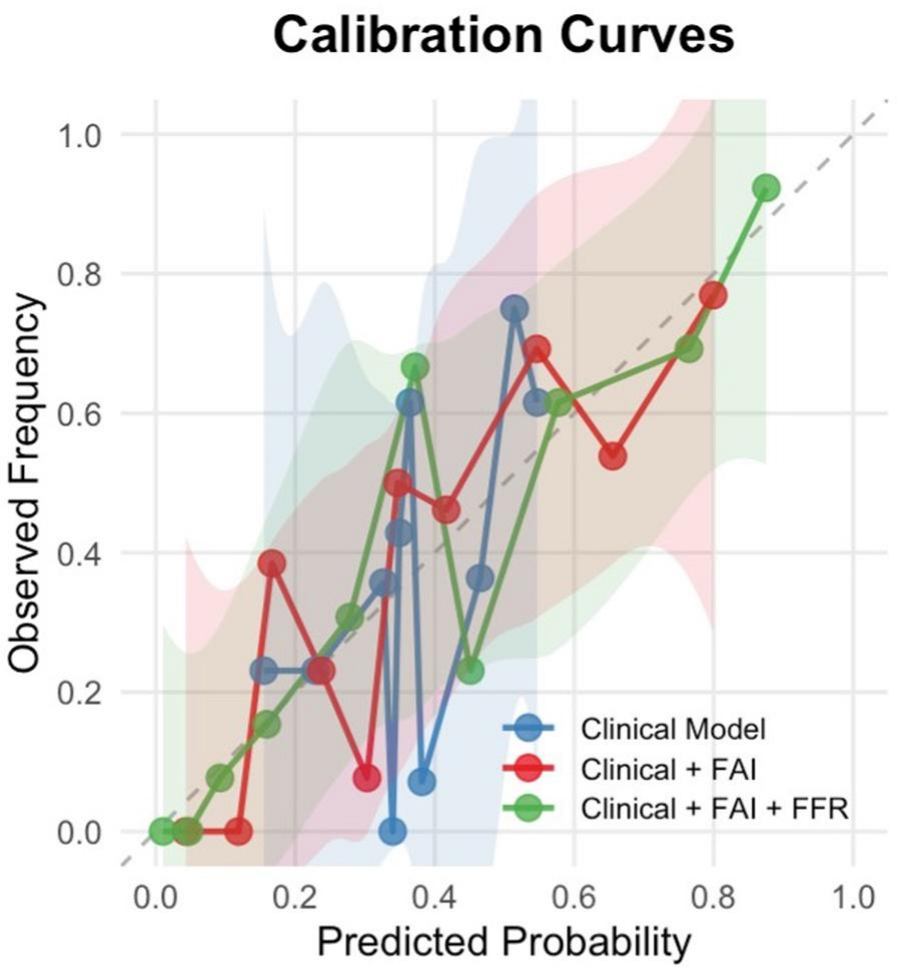
Calibration curves for restenosis prediction models. The calibration curves demonstrate the agreement between predicted probabilities and observed frequencies of restenosis across three predictive models:
Blue line: Clinical factors only (age, gender, hypertension, diabetes, smoking status)Red line: Clinical factors + Fat Attenuation Index (FAI)Green line: Clinical factors + FAI + Fractional Flow Reserve (FFR)The dashed diagonal line represents perfect calibration. Shaded areas indicate 95% confidence intervals estimated.

Calibration curve analysis revealed systematic overprediction by the base clinical model (blue curve), particularly in moderate-to-high risk ranges, where deviations reached 28% (Figure 6). Integration of the Fat Attenuation Index (FAI, red curve) substantially improved calibration accuracy, reducing maximum deviations to <10% in higher-risk strata (0.5–0.75). The multimodal CT-FAI-FFR model (green curve) demonstrated optimal calibration fidelity across the risk continuum, achieving <5% deviation in the clinically critical 0.5–0.75 probability range. Calibration analysis showed that the multimodal CT-FAI-FFR model (green curve) showed greater variability than the FAI enhancement model (red curve) in the probability interval of 0.3–0.45. This result may be due to the inherent variability of CT-FFR data or its interaction with clinical parameters and FAI is not fully captured.

## 4 Discussion

This study developed and validated the CT-FAI-FFR prediction model, a multimodal risk-stratification tool that provides fully automated, deep-learning-based integration of computed tomography-derived fractional flow reserve (CT-FFR) and pericoronary fat attenuation index (FAI) with a single, clinically validated software platform to predict in-stent restenosis (ISR). The combination of hemodynamic (CT-FFR) and inflammatory biomarkers (FAI) significantly improved ISR prediction beyond clinical factors alone (AUC 0.793 vs. 0.656, ΔAUC +0.137), supporting the notion that FAI and CT-FFR represent distinct yet complementary pathological pathways driving ISR.

A key advancement of our model is its clinically reliable calibration. The calibration curves (Figure 6) provide crucial insights beyond discrimination (AUC), specifically evaluating the accuracy of the predicted probabilities generated by our models. The observed systematic deviations highlight the limitations of relying solely on clinical factors for ISR prediction. The base clinical model (blue curve) exhibited significant overprediction, particularly in the moderate-to-high risk range. This overestimation suggests that models based purely on demographics and traditional risk factors lack precision and may lead to unnecessary anxiety or interventions in patients flagged as high-risk who, in reality, have a lower likelihood of ISR. The calibration curves demonstrate that incorporating FAI and FFR substantially improves model reliability. While the base clinical model showed systematic miscalibration (predicted probabilities > observed risks between 0.25–0.50 probability thresholds), the sequential addition of FAI (red curve) reduced this overestimation bias. Crucially, despite slight variation in medium-risk, the full model (Clinical + FAI + FFR, green curve) exhibited nearly optimal alignment with the ideal reference line across the entire risk spectrum. This indicates that predictions from the FAI-FFR integrated model accurately reflect true event probabilities.

The improved calibration stems from FFR's ability to counterbalance limitations of plaque imaging alone. FAI (reflecting inflammation) may overestimate risk in anatomically severe but functionally non-significant lesions, whereas FFR introduces physiological context (27). This synergy mitigates false-positive predictions, which is a critical advantage when selecting patients for intensive therapies.

The broad therapeutic window where the composite model maintained high net benefit (thresholds >0.20–0.70, spanning >50 probability points) suggests potential clinical utility, as clinicians could adjust intervention thresholds within this range without compromising predictive utility.

Our mixed-effects logistic regression analysis identified key predictors of in-stent restenosis (ISR) with strong clinical relevance. First, the Fat Attenuation Index (FAI) suggested an increased ISR risk (OR = 1.10, *p* = 0.021). Each 1-unit rise in FAI corresponded to a 10% higher ISR probability, confirming its value in measuring inflammation around stents. Prior studies indicated that perivascular adipose tissue inhibits pathological vascular remodeling through the secretion of anti-inflammatory factors such as pro-inflammatory substances, NO and adipokines. Chronic inflammation, metabolic disorder, or aging lead to PVAT dysfunction, promoting fibrosis adjacent to plaques (28). Recent studies focusing on new medications and stents have indicated that anti-inflammatory drugs and stents hold promise as a strategy to tackle in-stent restenosis (ISR) (29–32). Atherosclerotic plaques with more significant local inflammation are likely to derive greater benefits from these new drugs and stents. Pericoronary fat attenuation index (FAI) can be utilized to identify, before percutaneous coronary intervention (PCI), the plaques that require next-generation anti-inflammatory stents and the patients who need additional anti-inflammatory treatment to prevent ISR. 18F-fluorodeoxyglucose positron emission tomography/computed tomography (18F-FDG PET/CT) imaging is also capable of quantifying perivascular inflammation, but its clinical application is restricted by limited availability, high costs, and lasting radiation damage (33). In contrast, pericoronary FAI can overcome these drawbacks. It is quantified through extra post-processing of routine coronary computed tomography angiography (CCTA) without any additional radiation exposure, and CCTA is widely used in the non-invasive imaging of coronary artery disease.

A lower computed tomography-derived fractional flow reserve (CT-FFR) value was significantly associated with an increased risk of in-stent restenosis (ISR) (OR = 0.47 per 1-unit increase in FFR, 95% CI: 0.13–0.81, *p* = 0.041). This effect suggests blockages affecting blood flow (FFR ≤ 0.80) create low-stress zones accelerating scar tissue formation. Low CT-FFR values indicate significant hemodynamic stenosis, and abnormal shear stress triggers poor adaptive response. Some experiments have linked low shear stress with endothelial-mesenchymal transition and matrix metalloproteinase activation to drive the process of neointimal hyperplasia (11, 34, 35). It is worth noting that these pathways may interact: inflammatory cytokines could exacerbate microvascular dysfunction, while the hemodynamic impairment reflected by CT-FFR (often associated with altered shear stress) may, in turn, amplify the inflammatory response. This potential bidirectional interplay provides a plausible pathophysiological explanation for the superior predictive performance of the combined CT-FAI-FFR model.

In our study, smoking (OR = 1.71, *p* = 0.069) and BNP levels (OR = 0.98, *p* = 0.065) approached significance. Smokers faced 71% higher ISR risk, implying tobacco toxins intensify inflammation near stents. Higher BNP showed a modest protective trend (2% risk reduction per unit), hinting at compensatory effects limiting tissue remodeling—a hypothesis needing future study. Unexpectedly, in our full model, traditional factors such as diabetes and hypertension were not associated with ISR (*P* > 0.2). This suggests that the development of ISR depends more on local conditions (inflammation/blood flow) than systemic diseases.

High-sensitivity C-reactive protein (hsCRP) functions as an acute-phase reactant and a non-specific indicator of inflammation. A variety of factors can cause elevated hsCRP levels, such as infection, trauma, and atherosclerosis (36, 37). Due to these properties, hsCRP is unable to specifically reflect the local inflammatory status of coronary arteries. In our research, baseline hsCRP showed no correlation with the subsequent occurrence of in-stent restenosis (ISR), which aligns with findings from earlier studies (38, 39).

From a translational standpoint, this algorithm suggests a potential noninvasive approach for risk stratification before PCI. This hypothesizes a high-risk subgroup that might benefit from more intensive management strategies, and a low-risk subgroup in which invasive procedures might be deferred, thereby optimizing resource utilization.

This study has several limitations. Its single-center, retrospective design requires validation in future multi-center prospective cohorts. Although the exclusion of patients on high-dose statins or anti-inflammatory therapy was methodologically necessary to establish unbiased biomarker associations, it limits the model's immediate generalizability to real-world PCI populations. Furthermore, as a retrospective study, detailed data on long-term medication adherence and serial lipid profiles—important determinants of ISR risk—were not systematically collected; these unmeasured confounders should be addressed in future prospective validations. Additionally, the dependence on a proprietary software platform (Shukun) means the performance may vary, necessitating external validation with alternative systems. It is also important to note that the quantification of both FAI and CT-FFR can be influenced by technical factors such as CT tube voltage and reconstruction kernel, which represents a challenge for standardization across different imaging centers.

The range of follow-up durations (6–24 months) introduces potential variability in the timing of ISR detection, which could lead to outcome misclassification. Although our follow-up protocol was not symptom-triggered, which reduces ascertainment bias, the impact of variable follow-up times remains a consideration.

Finally, while our model demonstrated good performance in internal validation, the number of ISR events (*n* = 55) is modest relative to the number of predictors evaluated, raising a potential concern for overfitting. We employed statistical strategies to mitigate this risk, including mixed-effects modeling and repeated cross-validation. The stable performance observed in internal validation (cross-validated AUC = 0.781) is reassuring, but confirmation in a larger, independent cohort is essential to establish robust generalizability.

In conclusion, this study proposes the CT-FAI-FFR score as a multimodal, non-invasive predictor of in-stent restenosis (ISR). By integrating inflammatory activity (FAI) and hemodynamic significance (CT-FFR), the model demonstrates superior discriminative performance (AUC 0.793 vs. 0.656 for clinical factors alone, *p* < 0.05) and addresses critical gaps in existing ISR risk stratification. Key advances include the revelation of pathophysiological synergy—where FAI-quantified perivascular inflammation interacts with CT-FFR-detected low-shear stress to accelerate neointimal hyperplasia—and direct clinical translation through pre-PCI risk stratification using routine coronary CTA data. If validated prospectively, high-risk patients may benefit from personalized interventions: anti-inflammatory therapy, optimized stent selection in low-flow zones, or intensified surveillance. Crucially, this approach leverages widely available CCTA without added radiation or cost, thereby overcoming limitations of invasive angiography and PET. Furthermore, its exceptional calibration reliability suggests potential for clinical decision-making. Future multi-center studies should validate this tool in statin-treated cohorts and explore its utility for guiding post-PCI management.

## Data Availability

The raw data supporting the conclusions of this article will be made available by the authors, without undue reservation.
